# Microbiology challenges and opportunities in the circular economy

**DOI:** 10.1099/mic.0.001026

**Published:** 2021-01-25

**Authors:** Kevin E. O'Connor

**Affiliations:** ^1^​ BiOrbic Bioeconomy SFI Research centre and School of Biomolecular and Biomedical Science, University College Dublin, Dublin, Ireland

## Abstract

Our knowledge and understanding of micro-organisms have led to the development of safe food, clean water, novel foods, antibiotics, vaccines, healthier plants, animals and soils, and more, which feeds into the United Nations Sustainable Development Goals (UN SDGs). The circular economy can contribute to the UN SDGs and micro-organisms are central to circular nutrient cycles. The circular economy as described by the Ellen MacArthur foundation has two halves, i.e. technical and biological. On the technical side, non-biological resources enter manufacturing paths where resource efficiency, renewable energy and design extend the life of materials so that they are more easily reused and recycled. Biological resources exist on the other half of the circular economy. These are used to manufacture products such as bioplastics and paper. The conservation of nature’s stocks, resource efficiency and recycling of materials are key facets of the biological half of the circular economy. Microbes play a critical role in both the biological and technical parts of the circular economy. Microbes are key to a functioning circular economy, where natural resources, including biological wastes, are converted by microbes into products of value and use for society, e.g. biogas, bioethanol, bioplastics, building block chemicals and compost for healthy soils. In more recent times, microbes have also been seen as part of the tool kit in the technical side of the circular economy, where microbial enzymes can degrade plastics and microbes can convert those monomers to value-added products.

## Background

The United Nations Sustainable Development Goals (UN SDGs) are 17 global goals and 169 targets adopted by all UN Member States in September 2015. These targets follow on from the Millennium Development Goals, but have a greater breadth of activity and are substantially more ambitious. The goals aim to tackle major global challenges by 2030 and focus heavily on inequality and climate change. As scientists aiming to understand how things work and how we can exploit our knowledge to make the world a better place, many of us will feel we have an obligation to try and better focus our work in contributing to achieving some of the major challenges outlined in the UN SDGs. Unsurprisingly, health and the environment feature strongly. Many microbiologists globally are undertaking research to address grand challenges using innovative ideas and rigorous scientific methodologies. The ongoing coronavirus pandemic aptly exposes the threat that infectious disease poses to humankind, but also highlights how science can respond rapidly and collaboratively to generate novel methods to reduce the impact of a disease. But how do we approach such daunting tasks and what exciting knowledge do we have that can be applied directly to the UN SDGs?

The coronavirus disease 2019 (COVID-19) experience has been a lesson and a severe warning shot for the challenges we face and our preparedness for those challenges. It is increasingly apparent that the virus will be with us indefinitely and there will be other pandemics in the future that we will have to respond to with similar speed. We have to learn to work as a community, to generate knowledge that can be accessed by many so that it can be applied to address immediate and longer-term threats to humankind, such as pandemics, food security, environmental sustainability and antimicrobial resistance (AMR). The Microbiology Society recognizes these challenges and sees enormous potential in the microbiology research community in the UK and Ireland to jointly apply their skills in tackling these major global issues. Consequently, the society created an expansive project entitled ‘A Sustainable Future’ in 2019, which is working to define a principal role for microbiology in achieving some key aspects of the UN SDGs. This is an ongoing mission and the scope of the UN-defined goals is exceptionally broad and often interlocked with other development internationally; however, engagement with the society’s membership identified three specific areas where the expertise of microbiologists in the UK and Ireland is particularly relevant. These areas are AMR, which is widely recognized as one of the biggest threats to humanity; the circular economy, encompassing regenerative approaches aiming to maximize the most efficient use of the world’s finite resources; and soil health, which is essential for us to sustainably feed an ever-growing global population.

## The workshop

As opposed to dictating the agenda, as a society we have continuously sought to engage with the community as much as possible to direct ideas and try to focus efforts on the key expertise already held within its membership. As a component of this project, we held a week-long series of workshops in the summer of 2020 to identify challenges and opportunities for microbiology research and innovation, interdisciplinary collaboration, as well as interactions with industry, social and political institutions in the transition to a circular economy. As many of the raised concepts may not be feasible given current structures, we also asked what more could be done if there were fewer barriers, and what interventions would be needed to take the generated ideas to the next level.

Given that this workshop was performed during the ongoing pandemic, it was conducted online via focus groups chaired by recognized experts in each of the defined areas of the circular economy. The meeting chairs for each focus group selected two topic clusters that served to frame the discussion around challenges and opportunities for microbiology in the circular economy and how we could respond as a community. The clusters for each group were as follows. Microbiology research and innovation: (1) building a strong and resilient research community and (2) disseminating research. Interdisciplinary collaboration: (1) structures and funding and (2) managing expectations and internal communication. Industry: (1) research and industry collaboration and (2) policy, regulations and market. Social and political institution: (1) policy context and (2) engagement and culture.

## The discussion

The focus groups were purposely composed of people from a range of backgrounds, geographical locations and scientific seniority. The discussions were vibrant, occasionally passionate and generated a spectrum of opinions that could be directed into potential policy documents for how to both utilize and adapt the current microbiology research landscape in the UK and Ireland. Here, I outline some of the key thoughts and perceptions generated during these 90 min discussions. As a group we have tried to titrate these into some key concepts that may provide an insight into how the microbiology research community in the UK and Ireland can contribute to the circular economy transition.

### Microbiology research and innovation

Microbiologists working on microbial metabolism and transport, enzyme expression and engineering, microbial community interactions, biocatalysis, bioprocess engineering and more can directly generate knowledge and technology that can help to achieve circularity in society. While the focus on microbiology in the circular economy is often on waste valorization, microbes in soil contribute to soil health and thus also contribute to the circular economy by ensuring that the world has the platform to regenerate.

Microbiologists working in the circular economy field would benefit from collaboration, which would help to bring perspective to their research but also drive ideation on how their research and knowledge creation can be used for global challenges. This can also stimulate ideas on how to effectively communicate messages across to industry and wider society. More sophisticated bioinformatic tools allowing large-scale datasets analyses as well as training would help microbiologists think about the grand challenge earlier on.

Technology innovations have significant potential to accelerate the transition to the circular economy by combining digital, physical and biological technologies at scale. Scaling up microbial processes for commercial production requires time and investment. Microbiologists should seek to develop a network beyond microbiology and gain advice from those who have successfully executed scale-up.

Complex intellectual property (IP) agreements at university level are costly and time-consuming. Consortium agreements in the spirit of responsible partnering (such as the DESCA model in the EU) allow us to balance and protect the interests of all participant categories: universities, public research institutes, and large and small firms. A similar model, settled across the UK and Ireland, would provide more freedom and opportunities for microbiologists working in the circular economy field to partner up and share their work.

### Interdisciplinary collaboration

Open access infrastructures enabling interdisciplinary collaboration can be complex to manage and lack impact when operating in isolation from one another. There is a need for wider circular economy networks connecting dynamic infrastructures around a shared vision of what positive outcomes of interdisciplinary research will look like and how to get there.

Grand challenges and relevant approaches to address them evolve with time. Although the bioeconomy was initially envisaged as a linear replacement for a fossil-based economy, it now needs to be circular. Flexible funding deadlines with a clear plan and potential to work towards impact are required in order to allow projects to be dynamic/flexible and to allow new expertise to join projects, enabling teams to manage the challenges as they evolve.

The Microbiology Society could be involved in the creation of a virtual circular economy centre that would mentor and champion the development of an interdisciplinary culture and include social scientists, as grand challenges require both technical solutions and behavioural change.

### Industry

There is a need for a strategic framework that enables the development of a circular economy by allowing academia and industry to grow together and engage in the development of policy and regulation. Industry needs to be a partner from the very start in the design of approaches to grand challenges. This could be achieved through co-funding from industry and public funds, but there is a clear need to manage the expectations of all parties, as the drivers for such undertakings can be very different

The lack of resources in universities to manage IP portfolios can lead to the development of companies and the licensing of IP at a too early stage. There could be value in consolidating resources and focusing support to enable the transition from good ideas to viable businesses.

A physical or virtual space fostering non-competitive communication and showcasing the practical benefits and economic outcomes of successful circular economy projects is needed to encourage more research and industry collaboration.

### Social and political institutions

The circular economy will only be truly sustainable if it helps contribute to a transformation of society. From a policy point of view, major changes are needed in global, regional and national frameworks. Policies at different levels of government need to be aligned and work in harmony to advance meaningful progress. An ambitious systems approach, bringing different actors and systems together, is needed to bridge multiple contexts rather than relying on solutions generated in silos.

Greater society engagement is critical and could be gained by creating a coalition-building initiative similar to Climate Assembly UK. Such an assembly would bring together representatives of the population to hear balanced evidence on the choices the UK/Ireland face, discuss them, and make recommendations about what should be done in order to transition to a circular economy.

Most efforts to achieve a circular economy have so far centred on the ‘trash’ part of the circle, through recycling and waste management programmes. While this is important, there needs to be more focus on the ‘take’: reducing the amount of materials that we remove from nature to begin with.

### Closing remarks

Microbiology has a critical role to play in the transition of our society from a linear to a circular economy, which is interlinked with the challenge of achieving climate neutrality. Open and broad collaboration is essential to realizing change and impact. This brings with it many challenges, but the Microbiology Society can play a critical role in the structuring of these collaborations. Microbiologists must engage the wider public, policymakers and industry to inform the debate on addressing grand challenges and showcase the outputs, outcomes and impacts of microbes and microbiologists for society as a whole.

